# Validation of the ASDAS with a quick quantitative CRP assay (ASDAS-Q) in patients with axial SpA: a prospective multicentre cross-sectional study

**DOI:** 10.1177/1759720X221085951

**Published:** 2022-03-30

**Authors:** Fabian Proft, Julia Schally, Henning Christian Brandt, Jan Brandt-Juergens, Gerd Rüdiger Burmester, Hildrun Haibel, Henriette Käding, Kirsten Karberg, Susanne Lüders, Burkhard Muche, Mikhail Protopopov, Judith Rademacher, Valeria Rios Rodriguez, Murat Torgutalp, Maryna Verba, Silke Zinke, Denis Poddubnyy

**Affiliations:** Department of Gastroenterology, Infectiology and Rheumatology (including Nutrition Medicine), Charité – Universitätsmedizin Berlin, corporate member of Freie Universität Berlin and Humboldt-Universität zu Berlin, Hindenburgdamm 30, Berlin 12203, Germany; Department of Gastroenterology, Infectiology and Rheumatology (including Nutrition Medicine), Charité – Universitätsmedizin Berlin, corporate member of Freie Universität Berlin and Humboldt-Universität zu Berlin, Berlin, Germany; Praxis für Rheumatologie und Innere Medizin, Berlin, Germany; Rheumatologische Schwerpunktpraxis, Berlin, Germany; Department of Rheumatology and Clinical Immunology, Charité – Universitätsmedizin Berlin, corporate member of Freie Universität Berlin and Humboldt-Universität zu Berlin, Berlin, Germany; Department of Gastroenterology, Infectiology and Rheumatology (including Nutrition Medicine), Charité – Universitätsmedizin Berlin, corporate member of Freie Universität Berlin and Humboldt-Universität zu Berlin, Berlin, Germany; Department of Gastroenterology, Infectiology and Rheumatology (including Nutrition Medicine), Charité – Universitätsmedizin Berlin, corporate member of Freie Universität Berlin and Humboldt-Universität zu Berlin, Berlin, Germany; Praxis für Rheumatologie und Innere Medizin, Berlin, Germany; Department of Gastroenterology, Infectiology and Rheumatology (including Nutrition Medicine), Charité – Universitätsmedizin Berlin, corporate member of Freie Universität Berlin and Humboldt-Universität zu Berlin, Berlin, Germany; Department of Gastroenterology, Infectiology and Rheumatology (including Nutrition Medicine), Charité – Universitätsmedizin Berlin, corporate member of Freie Universität Berlin and Humboldt-Universität zu Berlin, Berlin, Germany; Department of Gastroenterology, Infectiology and Rheumatology (including Nutrition Medicine), Charité – Universitätsmedizin Berlin, corporate member of Freie Universität Berlin and Humboldt-Universität zu Berlin, Berlin, Germany; Department of Gastroenterology, Infectiology and Rheumatology (including Nutrition Medicine), Charité – Universitätsmedizin Berlin, corporate member of Freie Universität Berlin and Humboldt-Universität zu Berlin, Berlin, Germany; Berlin Institute of Health (BIH), Berlin, Germany; Department of Gastroenterology, Infectiology and Rheumatology (including Nutrition Medicine), Charité – Universitätsmedizin Berlin, corporate member of Freie Universität Berlin and Humboldt-Universität zu Berlin, Berlin, Germany; Department of Gastroenterology, Infectiology and Rheumatology (including Nutrition Medicine), Charité – Universitätsmedizin Berlin, corporate member of Freie Universität Berlin and Humboldt-Universität zu Berlin, Berlin, Germany; Department of Gastroenterology, Infectiology and Rheumatology (including Nutrition Medicine), Charité – Universitätsmedizin Berlin, corporate member of Freie Universität Berlin and Humboldt-Universität zu Berlin, Berlin, Germany; Rheumapraxis Berlin, Berlin, Germany; Department of Gastroenterology, Infectiology and Rheumatology (including Nutrition Medicine), Charité – Universitätsmedizin Berlin, corporate member of Freie Universität Berlin and Humboldt-Universität zu Berlin, Berlin, Germany; Epidemiology Unit, German Rheumatism Research Centre, Berlin, Germany

**Keywords:** ankylosing spondylitis, disease activity, spondyloarthritis, treat-2-target – ASDAS

## Abstract

**Objectives:**

The objective of the study was to validate the Ankylosing Spondylitis Disease Activity Score (ASDAS) based on a quick quantitative C-reactive protein (qCRP) assay (ASDAS-Q) in a multicentre, prospective, cross-sectional study in patients with axial spondyloarthritis (axial SpA).

**Methods:**

Disease activity assessment was performed in prospectively recruited patients with axial SpA. Routine laboratory CRP was determined in the central laboratory of each study centre, while quick qCRP and erythrocyte sedimentation rate (ESR) were measured locally. Consequently, ASDAS-CRP, ASDAS-Q using the qCRP and ASDAS-ESR were calculated. The absolute agreement on the disease activity category ascertainment was analysed with cross-tabulations and weighted Cohen’s kappa. Bland–Altman plots and intraclass correlation coefficients (ICCs) were used to analyse the criterion validity.

**Results:**

Overall, 251 axial SpA patients were included in the analysis. The mean qCRP value (6.34 ± 11.13 mg/l) was higher than that of routine laboratory CRP (5.26 ± 9.35 mg/l). The ICC for routine laboratory CRP *versus* qCRP was 0.985 [95% confidence interval (CI): 0.972–0.991]. Comparing ASDAS-Q with ASDAS-CRP, 242 of 251 (96.4%) patients were assigned to the same disease activity categories with a weighted Cohen’s kappa of 0.966 (95% CI: 0.943–0.988) and ICC of 0.997 (95% CI: 0.994–0.999).

**Conclusions:**

ASDAS-Q showed an almost perfect agreement with ASDAS-CRP in the assignment to specific disease activity categories. Consequently, ASDAS-Q using the qCRP value can be applied as an accurate and quickly available alternative to ASDAS-CRP, thus facilitating the implementation of the treat-to-target concept in clinical trials and clinical routine.

## Key messages

- This study validated the ASDAS based on a quick quantitative CRP assay (ASDAS-Q).
- Agreement of disease activity category assignment by ASDAS-CRP and ASDAS-Q was observed in 96%.
- The ASDAS-Q may help to implement the T2T concept in clinical trials and clinical practice.

## Introduction

Axial spondyloarthritis (axial SpA) is a common rheumatic disease and typically manifests in young adulthood.^1,2^ It predominantly causes inflammation of sacroiliac joints and spine resulting in chronic back pain.^3^ Axial SpA patients may also suffer from peripheral manifestations like enthesitis, peripheral arthritis and dactylitis as well as extra-musculoskeletal manifestations (EMMs), such as uveitis, psoriasis and inflammatory bowel disease.^3,4^ Axial SpA can be divided into two subgroups: nonradiographic axial SpA and radiographic axial SpA (r-axial SpA) – also called ankylosing spondylitis (AS).^3^ The distinction of both forms relies on the absence or presence of definite radiographic sacroiliitis according to the modified New York criteria.^5,6^

Regular assessment of disease activity and therapeutic decision-making based on this assessment are key aspects in rheumatologic patient care^7^ as reflected in the treat-to-target (T2T) concept.^8^ The Ankylosing Spondylitis Disease Activity Score (ASDAS)^9,10^ is the recommended score for disease activity assessment in axial SpA according to international guidelines.^8,11^ Achieving ‘inactive disease’ according to the ASDAS was associated with significantly more pronounced improvements in physical function, work productivity and health-related quality of life (hr-QoL) in axial SpA patients.^12,13^ Furthermore, persistently high ASDAS values were significantly associated with a higher chance of new syndesmophyte formation and accelerated radiographic spinal progression.^14–16^ These observations highlight the prognostic importance of regular ASDAS assessments and suggest an improved outcome when applying ASDAS-driven therapeutic decisions.

In addition to patient-reported outcomes (PROs), ASDAS incorporates acute phase reactants [C-reactive protein (CRP) or erythrocyte sedimentation rate (ESR)] as objective parameters of disease activity. According to international expert recommendations, the CRP-based ASDAS (ASDAS-CRP) is the preferred tool for disease activity assessment in axial SpA.^10,11^ Determination of routine laboratory CRP, however, takes several hours to days, which causes a time delay in ASDAS calculation using routine laboratory CRP values. This hinders the widespread use of ASDAS for disease activity assessment despite the evidence supporting its benefits and complicates the implementation of the T2T concept in the outpatient setting and also in clinical trials.

An ASDAS assessment using ESR measurement (ASDAS-ESR) still takes at least 1 h and is more difficult to perform in a standardized way, which is particularly disadvantageous in clinical trials.^10^ Moreover, high ESR values seem to be less specific for systemic inflammation in axial SpA compared with increased CRP values.^15,17,18^

Quick quantitative CRP (qCRP) assays can be performed within a few minutes and hence a qCRP-based ASDAS (ASDAS-Q) might be a suitable option for disease activity assessment in axial SpA patients with an immediate result. In a single-centre, cross-sectional pilot study, a high level of agreement on disease activity categories assignment by ASDAS-Q compared with ASDAS-CRP (94%) in 50 newly diagnosed, biologic-naïve axial SpA patients was shown.^19^ The aim of our current project was to validate the ASDAS-Q for the assessment of disease activity in a prospective, multicentre, cross-sectional study in a broad population of axial SpA patients.

## Methods

### Patients and assessments

This cross-sectional, multicentre study was conducted in Berlin, Germany. Five rheumatologic centres (two specialized academic university hospitals and three rheumatologic outpatient practices) participated in this project. Participants were enrolled between January 2020 and September 2020. All adult (⩾18 years) patients with a clinical diagnosis of axial SpA, who had given written informed consent, were considered eligible for study participation. Rheumatologists were encouraged to include consecutive patients. Subjects with a known clinically significant anaemia, defined as haemoglobin (Hb) concentration <10 g/dl, or with signs of an acute infection were excluded. After providing written informed consent, eligible patients underwent a structured rheumatologic assessment, including patients’ history, physical examination, PROs [Bath Ankylosing Spondylitis Disease Activity Index (BASDAI) questionnaire^20^ and patient global assessment of disease activity on a numeric rating scale (0–10)] and laboratory assessment. Routine laboratory CRP was measured by a conventional method (usually, a turbidimetric assay) in different certified laboratories. ESR values were collected locally. All qCRP measurements were carried out with the ‘QuikRead go’ instrument (software versions 7.5.1 and 7.6.2.; Aidian Oy, Espoo, Finland) and the reagent kits ‘QuikRead go wrCRP + Hb’ (Aidian Oy). Mostly, qCRP measurements could be performed with whole blood from the blood collection system, which would have been disposed otherwise. When the blood in the collection system was already coagulated or the collection system was not available for the analysis, ethylenediaminetetraacetic acid (EDTA) or heparin whole blood could be used for qCRP analysis instead. Measurement range of qCRP values depended on the haematocrit concentration and was 0.5–200 mg/l for a haematocrit concentration of 40–45%.^21^ Validity studies for the ‘QuikRead go’ products were done according to the Clinical and Laboratory Standards Institute (CLSI) guidelines, EP5-A3.^21,22^ The qCRP measurement was performed according to the assay instructions.^21^ All qCRP values were indicated with one decimal place by the qCRP measurement device. All routine laboratory CRP values were indicated with one decimal place with the exception of two patients, in which the routine laboratory CRP was indicated with two decimal places by the laboratory.

Quality controls were performed once weekly with ‘QuikRead go wrCRP Control’ (Aidian Oy) to ensure correct measurement and reduce the risk of bias. In addition, the multicentre design involving qCRP measurements at different locations and routine laboratory CRP measurement in different central laboratories contributed to reducing the risk of bias.

After all needed parameters were available, disease activity scores (ASDAS-CRP, ASDAS-Q and ASDAS-ESR^9,10^) were calculated based on routine laboratory CRP value, qCRP value and ESR. If the CRP or qCRP value was below the lowest limit of detection, a value of 0.0 mg/l was assumed. If the CRP or qCRP value was <2 mg/l, the fixed value of 2 mg/l was used for the calculation of the ASDAS-CRP and ASDAS-Q, respectively, as recommended by Machado *et al.*^23^ The ASDAS-CRP and ASDAS-Q values were obtained with the official Assessment of Spondyloarthritis international Society (ASAS) calculator. This calculator delivered ASDAS-CRP and ASDAS-Q values with only one decimal place. ASDAS-ESR values were calculated according to the formula by Machado *et al.*^23^ For the analysis of identical numerical ASDAS values (see ‘Outcomes’ section), ASDAS-ESR values were rounded to one decimal place.

Patients were assigned to the following disease activity categories according to the values of ASDAS-CRP, ASDAS-Q and ASDAS-ESR: inactive disease = ASDAS < 1.3; low disease activity = ASDAS ⩾ 1.3 and <2.1; high disease activity = ASDAS ⩾ 2.1 and ⩽ 3.5; and very high disease activity = ASDAS > 3.5.^24,25^

### Outcomes

The primary outcome of this study was the proportion of patients with identical disease activity category assignment by ASDAS-CRP and ASDAS-Q. ‘Optimal agreement’ between ASDAS-CRP and ASDAS-Q was prespecified at a level of agreement of at least 90% between the disease activity categories by ASDAS-CRP and ASDAS-Q.

Secondary outcome measures were the following:

- The proportion of identical numerical values for ASDAS-CRP and ASDAS-Q.
- The proportion of patients with identical disease activity categories by ASDAS-CRP and ASDAS-ESR.
- The proportion of identical numerical values for ASDAS-CRP and ASDAS-ESR.
- The proportion of identical numerical values for qCRP and routine laboratory CRP.

Identical numerical ASDAS and CRP/qCRP values were assumed if the numerical values were matching up to the first decimal place. For the analysis of identical numerical values, ASDAS-ESR values and routine laboratory CRP values (with more than one decimal place) were rounded to one decimal place. A proportion of identical numerical values in >50% of patients would be expected as ‘good agreement’.

### Sample size

For the sample size calculation, we used the proportion of patients who can be classified differently in terms of ASDAS disease activity categories as a result of ASDAS quick CRP calculation as compared with ASDAS routine CRP as a target outcome.

We assume that measurement of routine CRP with different labs/different assays would result in a different classification in about 5% of the patients. We also assume that up to 10% of re-classification based on ASDAS-Q as compared with ASDAS based on the routine CRP method would still be clinically acceptable. We would therefore need *n* = 239 patients to demonstrate a noninferiority of the ASDAS quick CRP as compared with ASAS routine CRP with a power of 90% using the *Z*-test for binominal proportions.

### Statistical analysis

Methods of descriptive statistics were applied for demographic data, clinical and laboratory information, and disease activity scores. The intake of nonsteroidal anti-inflammatory drugs (NSAIDs) was analysed by the calculation of the NSAID equivalent score (modified formula according to Dougados *et al.*^26^):

$$\begin{array}{l} \mathrm{NSAID}\;\mathrm{equivalent}\;\mathrm{score}\;=\;\\ \mathrm{NSAID}\;\mathrm{equivalent}\;\mathrm{dose}\;\;\times \frac{\mathrm{days}\;\mathrm{of}\;\mathrm{in}\;\mathrm{take}\;\mathrm{per}\;\mathrm{week}}{7} \end{array}$$

For the comparison of disease activity categories, cross-tabulations and weighted Cohen’s kappa were performed for ASDAS-CRP and ASDAS-Q as well as ASDAS-CRP and ASDAS-ESR. The agreement between the numerical values of different disease activity scores and between CRP *versus* qCRP was analysed using Bland–Altman plots and intraclass correlation coefficient (ICC); ICC and their 95% confidence intervals (CIs) were calculated with a mean-rating (*k* = 2), absolute agreement, two-way mixed-effects model. All statistical analyses were performed with SPSS Statistics (version 27; IBM, Armonk, NY, USA) and Microsoft Excel (Office 2019; Microsoft Corporation, Redmond, Washington, USA).

Patients with missing routine laboratory CRP or qCRP values were completely excluded from the statistical analysis. Patients with missing ESR values (and consequently also missing ASDAS-ESR values) were generally included in the statistical analysis, but all outcomes regarding ESR or ASDAS-ESR values were only investigated for patients having available ESR and ASDAS-ESR values.

### Ethical approval

This study was conducted in accordance with the ethical principles of the Declaration of Helsinki^27^ and Good Clinical Practice (GCP).^28^ The responsible ethical committee of the coordinating study centre (Charité – Universitätsmedizin Berlin, Berlin, Germany) approved the study in advance (EA4/208/19). All patients provided written informed consent before any study-specific procedures were performed. The reporting of this study confirms to the Strengthening the Reporting of Observational Studies in Epidemiology (STROBE) statement.^29^

### Funding statement

This work was partially supported by an unrestricted research grant from Novartis [MAIN457A_FVMH0]. Testing kits were provided free of charge from Aidian Oy.

## Results

### Demographic and clinical characteristics

Altogether, 253 axial SpA patients were eligible and have given written informed consent for study participation. Two of these patients could not be included in the statistical analysis: One patient could not be included in the statistical analysis because of a missing routine laboratory CRP value due to an incorrect laboratory order; the other patient had a missing qCRP value due to a mistake in the qCRP measurement. The knowledge of both routine laboratory CRP and qCRP values was necessary to analyse the primary outcome measure. A total of 251 axial SpA patients were included in the statistical analysis with routine laboratory CRP and qCRP values being available, while ESR measurement and ASDAS-ESR were available for 243 of those patients.

Clinical, demographic and treatment data are shown in Table 1. Mean age of the included patients was 38.4 years; mean symptom duration was 6.2 years; 159 (63.3%) patients were male; 211 (84.1%) were positive for human leukocyte antigen-B27 (HLA-B27) and 195 (77.7%) patients had r-axial SpA. For two patients, HLA-B27 status was not available.

**Table 1.** Demographic, clinical, laboratory and treatment characteristics.

Demographics	
Age in years, mean ± SD	38.4 ± 11.4
Disease duration in years, mean ± SD	6.2 ± 7.1
Male sex, *n* (%)	159 (63.3)
Imaging and laboratory	
r-axial SpA, *n* (%)	195 (77.7)
HLA-B27 positive, *n* (%)	211 (84.1)^a^
Routine laboratory CRP in mg/l, mean ± SD	5.3 ± 9.4
qCRP in mg/l, mean ± SD	6.3 ± 11.1
Difference between routine laboratory CRP and qCRP in mg/l, mean ± SD	1.1 ± 2.3
ESR in mm/h, mean ± SD	15.2 ± 16.3
Disease activity	
BASDAI, mean ± SD	3.2 ± 2.1
ASDAS-CRP, mean ± SD	2.1 ± 1.0
ASDAS-Q, mean ± SD	2.2 ± 1.0
ASDAS-ESR, mean ± SD	2.1 ± 1.1
Treatment	
NSAIDs, *n* (%)	135 (53.8)
Mean NSAID equivalent score ± SD for patients receiving NSAIDs^b^	58.4 ± 40.7
Number of patients with NSAID equivalent score ⩾ 100^b^	53 (21.1)
bDMARDs, *n* (%)	143 (57.0)
TNF inhibitors, *n* (%)	124 (49.4)
IL-17 inhibitors, *n* (%)	17 (6.8)
IL-12/-23 inhibitors, *n* (%)	2 (0.8)
csDMARDs, *n* (%)^c^	7 (2.8)
Systemic GC, *n* (%)^d^	6 (2.4)
NSAIDs mono-therapy, *n* (%)	82 (32.7)
bDMARDs mono-therapy, *n* (%)	90 (35.9)
NSAIDs + bDMARDs combination, *n* (%)	53 (21.1)
No NSAIDs and no bDMARDs, *n* (%)	26 (10.4)

ASDAS, Ankylosing Spondylitis Disease Activity Score; BASDAI, Bath Ankylosing Spondylitis Disease Activity Index; bDMARDs, biological DMARDs; CRP, C-reactive protein; csDMARDs, conventional synthetic DMARDs; DMARDs, disease-modifying anti-rheumatic drugs; ESR, erythrocyte sedimentation rate; GC, glucocorticosteroids; HLA-B27, human leukocyte antigen B27; IL, interleukin; NSAIDs, nonsteroidal anti-inflammatory drugs; qCRP, quick quantitative CRP; r-axial SpA, radiographic axial spondyloarthritis; SD, standard deviation; TNF, tumour necrosis factor; tsDMARDs, targeted synthetic DMARDs.

*n* = 251 for ESR and *n* = 243 for ASDAS-ESR.

a For two patients, HLA-B27 status was not available.

b Modified formula for NSAID equivalent score based on Dougados *et al*.^26^: NSAID equivalent dose × (days of intake per week/7).

c Five (2.0%) patients received sulfasalazine and two (0.8%) patients methotrexate.

d Mean dosage of prednisolone equivalent ± *SD* for patients under GC was 14.5 ± 18.5 mg/d.

In this study, 82 (32.7%) patients were treated with NSAID mono-therapy, 90 (35.9%) patients received biological disease-modifying anti-rheumatic drugs (bDMARDs) only and 53 (21.1%) patients were treated with a combination of NSAIDs and bDMARDs, while 26 (10.4%) patients received neither NSAIDs nor bDMARDs at the time of study participation. In total, 135 (53.8%) patients received an NSAID therapy with a mean NSAID equivalent score of 58.4 ± 40.7 and 53 (21.1%) patients showed an NSAID equivalent score ⩾ 100.

### Comparison of routine laboratory CRP and qCRP

**Figure 1.**
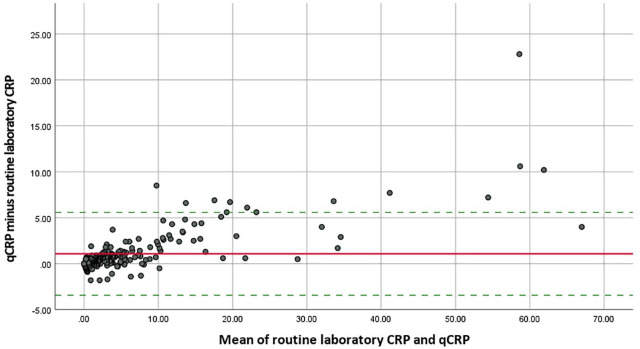
Bland–Altman plot for routine laboratory CRP and qCRP. CRP, C-reactive protein; qCRP, quick quantitative CRP. The solid line marks the mean difference between routine laboratory CRP and qCRP (1.081). The dotted lines indicate the 95% limits of agreement (–3.421 to 5.582).

The mean values of the routine laboratory CRP and qCRP were 5.3 ± 9.4 mg/l and 6.3 ± 11.1 mg/l, respectively (Table 1). Identical values by routine laboratory CRP and qCRP were observed in 35 of 251 (13.9%) patients. The mean difference of both CRP measurement methods was 1.1 ± 2.3 mg/l with differences ranging from −1.8 to 22.8 mg/l. The agreement of both CRP methods is depicted by the Bland–Altman plot (Figure 1).

The ICC for routine laboratory CRP and qCRP was 0.985 (95% CI: 0.972–0.991) (Table 2).

**Table 2.** ICC for different CRP measurement methods and ASDAS.

	ICC	95% confidence interval
Routine laboratory CRP and qCRP (*n* = 251)	0.985	0.972–0.991
ASDAS-CRP and ASDAS-Q (*n* = 251)	0.997	0.994–0.999
ASDAS-CRP and ASDAS-ESR (*n* = 243)	0.962	0.951–0.970
Model: two-way mixed-effects model Type: multiple measurements Definition: absolute agreement		

ASDAS, Ankylosing Spondylitis Disease Activity Score; CRP, C-reactive protein; ESR, erythrocyte sedimentation rate; qCRP, quick quantitative CRP; ICC, intraclass correlation coefficient.

### Comparison of ASDAS-CRP, ASDAS-Q and ASDAS-ESR

The ASDAS-Q provided the same assignment to a disease activity category as the ASDAS-CRP in 242 of 251 (96.4%) patients with a weighted Cohen’s kappa of 0.966 (95% CI: 0.943–0.988) (Table 3). Nine patients (3.6%) differed in their disease activity category by ASDAS-Q in comparison with ASDAS-CRP; with all nine being assigned to a higher disease activity category by ASDAS-Q than by ASDAS-CRP. Four of these nine patients had an ASDAS-CRP value of 2.0 and an ASDAS-Q value of 2.1, which represent the threshold between low and high disease activities.

**Table 3.** Disease activity categories by (A) ASDAS-Q *versus* ASDAS-CRP and (B) by ASDAS-CRP *versus* ASDAS-ESR.

**A**		**ASDAS-Q (*n* = 251)**			
		**Inactive disease** (<1.3)	**Low disease activity** (⩾1.3 and <2.1)	**High disease activity** (⩾2.1 and ⩽3.5)	**Very high disease activity** (>3.5)
**ASDAS-CRP**	**Inactive disease** (<1.3)	**56 (22.3%)**	**2 (0.8%)**		
	**Low disease activity** (⩾1.3 and <2.1)		**62 (24.7%)**	**7 (2.8%)**	
	**High disease activity** (⩾2.1 and ⩽3.5)			**97 (38.6%)**	
	**Very high disease activity** (>3.5)				**27 (10.8%)**
Weighted Cohen’s kappa: 0.966 (95% CI: 0.943–0.988)					
**B**		**ASDAS-ESR (*n* = 243)**			
		**Inactive disease** (<1.3)	**Low disease activity** (⩾1.3 and <2.1)	**High disease activity** (⩾2.1 and ⩽3.5)	**Very high disease activity** (>3.5)
**ASDAS-CRP**	**Inactive disease** (<1.3)	**48 (19.8%)**	**9 (3.7%)**		
	**Low disease activity** (⩾1.3 and <2.1)	**17 (7.0%)**	**42 (17.3%)**	**6 (2.5%)**	
	**High disease activity** (⩾2.1 and ⩽3.5)	**1 (0.4%)**	**15 (6.2%)**	**70 (28.8%)**	**8 (3.3%)**
	**Very high disease activity** (>3.5)			**7 (2.9%)**	**20 (8.2%)**
Weighted Cohen’s kappa: 0.756 (95% CI: 0.701–0.811)					

ASDAS, Ankylosing Spondylitis Disease Activity Score; CI, confidence interval; CRP, C-reactive protein; ESR, erythrocyte sedimentation rate; qCRP, quick quantitative CRP.

Fields highlighted in red indicate that disease activity categories do not match. Percentage values refer to 251 patients in the comparison of ASDAS-CRP *versus* ASDAS-Q and to 243 patients in the comparison of ASDAS-CRP *versus* ASDAS-ESR.

The deviation of disease activity categories between ASDAS-CRP and ASDAS-Q never exceeded more than one disease activity category (meaning inactive disease *versus* low disease activity and low disease activity *versus* high disease activity) (Supplementary Table S1).

Identical numerical values of ASDAS-Q and ASDAS-CRP were observed in 136 of 251 (54.2%) patients. The mean difference between ASDAS-Q and ASDAS-CRP was 0.05 ± 0.09 with observed differences ranging from −0.3 to 0.5. ICC for ASDAS-Q and ASDAS-CRP was 0.997 (95% CI: 0.994–0.999) (Table 2).

**Figure 2.**
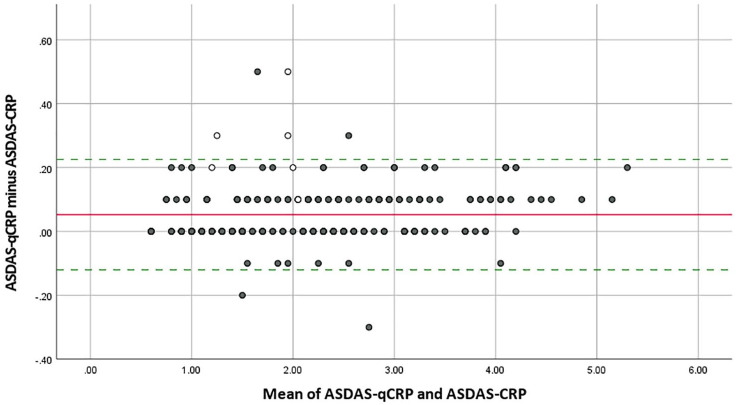
Bland–Altman plot for ASDAS-Q and ASDAS-CRP. ASDAS, Ankylosing Spondylitis Disease Activity Score; CRP, C-reactive protein; qCRP, quick quantitative CRP. Difference in disease activity category between ASDAS-Q and ASDAS-CRP: • Same disease activity category ○ Higher disease activity category with ASDAS-Q (one category higher with ASDAS-Q) The solid line marks the mean difference between ASDAS-Q and ASDAS-CRP (0.053). The dotted lines indicate the 95% limits of agreement (–0.120 to 0.226).

The agreement of ASDAS-Q and ASDAS-CRP values is graphically presented in a Bland–Altman plot (Figure 2).

When ASDAS-CRP was compared with ASDAS-ESR, 180 of 243 (74.1%) patients were assigned to the same disease activity categories with a weighted Cohen’s kappa of 0.756 (95% CI: 0.701–0.811) as shown in Table 3. Within the 63 (25.9%) patients assigned to different disease activity categories by ASDAS-CRP and ASDAS-ESR, 40 patients had a lower disease activity category by ASDAS-ESR with one of these patients showing a difference of two disease activity categories (inactive disease by ASDAS-ESR *versus* high disease activity by ASDAS-CRP). Discrepancies of ASDAS-ESR and ASDAS-CRP were observed in all disease activity categories.

**Figure 3.**
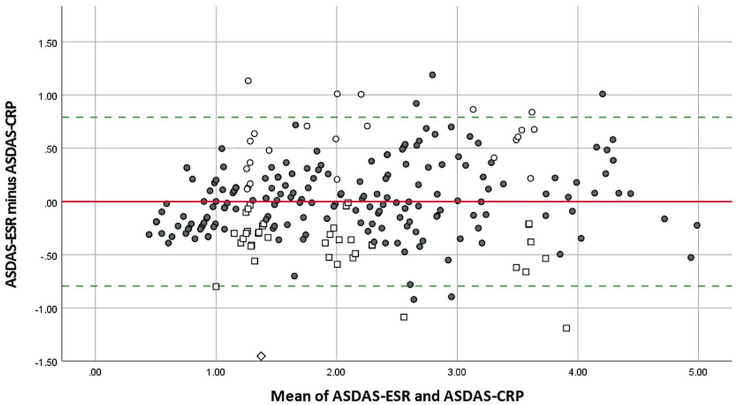
Bland–Altman plot for ASDAS-ESR and ASDAS-CRP. ASDAS, Ankylosing Spondylitis Disease Activity Score; CRP, C-reactive protein; ESR, erythrocyte sedimentation rate. Difference in disease activity category between ASDAS-ESR and ASDAS-CRP: ◇ Higher disease activity category with ASDAS-CRP (two categories higher with ASDAS-CRP) □ Higher disease activity category with ASDAS-CRP (one category higher with ASDAS-CRP) • Same disease activity category ○ Higher disease activity category with ASDAS-ESR (one category higher with ASDAS-ESR) The solid line marks the mean difference between ASDAS-ESR and ASDAS-CRP (–0.001). The dotted lines indicate the 95% limits of agreement (–0.794 to 0.791).

The ASDAS-ESR provided identical numerical values as the ASDAS-CRP in 29 of 243 (11.9%) patients when ASDAS-ESR values were rounded to one decimal. The mean of the differences between ASDAS-ESR and ASDAS-CRP was −0.001 ± 0.40 with observed differences ranging from −1.45 to 1.19. ICC for ASDAS-CRP and ASDAS-ESR was 0.962 (95% CI: 0.951–0.970) as presented in Table 2. A Bland–Altman plot for ASDAS-ESR and ASDAS-CRP illustrates the agreement of numerical values (Figure 3).

## Discussion

In the current study, we investigated the performance of ASDAS based on a previously validated qCRP assay in comparison with ASDAS calculated with routine laboratory CRP. Within a few minutes after blood drawing, qCRP measurement can be performed with a point-of-care technique, making ASDAS-Q a timely available disease activity score. Furthermore, qCRP measurement only requires approximately one drop of blood, which can be simply obtained from the fingertip and does not require venous blood sampling. ASDAS-Q showed an almost perfect agreement with ASDAS-CRP in the assignment to disease activity categories (>96%) indicating that ASDAS-Q can be applied as a feasible and accurate alternative to ASDAS based on routine laboratory CRP in axial SpA patients. The study cohort represents a typical axial SpA cohort as 63% were male, the average age was below 40 years, more than 80% were HLA-B27 positive,78% showed r-axial SpA and the included patients were treated with a broad spectrum of available therapeutics for axial SpA.

The proportion of patients with identical disease activity categories by ASDAS-CRP and ASDAS-Q is the most important parameter to evaluate the validity and clinical usefulness of the ASDAS-Q, since the disease activity category of a patient implicates the need for therapeutic changes and not a certain ASDAS value itself. Therefore, this proportion was chosen as primary outcome measure of the study. The analysis of identical numerical values by routine laboratory CRP/qCRP, ASDAS-CRP/ASDAS-Q and ASDAS-CRP/ASDAS-ESR provides an additional measure of agreement besides the agreement for disease activity categories. Although the analysis of identical numerical values is clinically less relevant than the analysis of identical disease activity categories, these secondary outcome measures help to better assess the validity of qCRP and ASDAS-Q in comparison with routine laboratory CRP and ASDAS-CRP. We decided against choosing a certain level of agreement for the numerical values as secondary outcome since we could not define a clear cut-off value which deviation would be clinically acceptable and which would not. For example, a small difference of 0.1 between ASDAS-CRP and ASDAS-Q could assign a patient to different disease activity categories when ASDAS-CRP is 2.0 (low disease activity) and ASDAS-Q is 2.1 (high disease activity). On the contrary, a large difference of two full points and more would not be clinically important when the patient is already assigned to the ‘very high disease activity’ category (e.g. ASDAS-CRP of 4.0 and ASDAS-Q of 6.0). Therefore, we simply chose the proportion of identical numerical values as a secondary outcome measure.

Identical numerical values by routine laboratory CRP and qCRP were observed in 13.9%. In this analysis, qCRP showed a tendency for higher values than routine laboratory CRP with a mean difference of 1.08 mg/l. This may explain the relatively small proportion of identical numerical values. In contrast to our pilot study,^19^ the lowest detection limit of the used qCRP assays was 0.5 mg/l, which is similar compared with most routine laboratory CRP assays In the pilot study, we observed a slightly higher difference of 1.39 mg/l, which may be explained by notably different lowest detection limits and corresponding replacement (qCRP: <5 mg/l replaced with 2 mg/l; routine laboratory CRP: <0.3 mg/l replaced with 0 mg/l).^19^ The tendency for higher qCRP values was especially observed for generally high CRP values as visualized in the Bland–Altman plot (Figure 1): Above the threshold of 10 mg/l, qCRP always showed a higher numerical value than routine laboratory CRP. The tendency for higher qCRP values, however, only had a minimal effect on the assignment of disease activity categories as ASDAS-CRP and ASDAS-Q showed a very high agreement with respect to identical disease activity categories. A possible explanation might be that axial SpA patients with high CRP values mainly showed ‘high’ or ‘very high disease activity’ according to ASDAS, where minor deviations of the absolute CRP value only had a small effect on disease activity assignment. Furthermore, in patients with ‘very high disease activity’ the absolute numerical ASDAS value should not have a relevant clinical impact, as all patients of this group would require escalation of their therapy when correctly applying the T2T concept. Thus, the tendency of slightly higher qCRP values and the relatively small proportion of identical values by routine laboratory CRP and qCRP do not seem to have a significant clinical relevance when qCRP is used for ASDAS calculation in axial SpA patients.

The ASDAS-Q clearly outperformed the ASDAS-ESR in terms of agreement with the ‘gold standard’ ASDAS-CRP. More than 25% of the patients were assigned to a different disease activity category by ASDAS-ESR – which is in line with previous literature19 – while this occurred only in 3.6% when using the ASDAS-Q – all of them were assigned to a higher disease activity category with ASDAS-Q reflecting the tendency of higher qCRP values compared with routine laboratory CRP. Furthermore, ASDAS-Q and ASDAS-CRP showed identical numerical values in more than half of the patients which applies to our pre-specified limits of ‘good agreement’. Again, these observations show that the differences between routine laboratory CRP and qCRP mostly did not have a relevant clinical impact as ASDAS-CRP and ASDAS-Q showed ‘optimal agreement’ regarding identical disease activity categories and ‘good agreement’ regarding identical numerical values. The agreement of numerical values between ASDAS-CRP and ASDAS-ESR was rather low which is in line with the relatively high disagreement of disease activity categories between ASDAS-CRP and ASDAS-ESR.

Two international treatment and disease management recommendations have given a clear preference for applying the T2T concept in axial SpA,^8,11^ while others conditionally voted against the application of the T2Tconcept aimed at certain ASDAS values.^30^ This discrepancy is mainly due to the fact, that only indirect evidence favouring the T2T concept in axial SpA^12–16,31^ is available.

Randomized controlled clinical trials comparing the T2T concept *versus* standard of care (SOC) in axial SpA patients have already published their first results (TICOSPA trial,^32^ NCT03043846), are currently ongoing (AScalate trial,^33^ NCT03906136) or were terminated prematurely because of slow recruitment (STRIKE trial, NCT02897115). The TRACE trial (NCT03639740) is an interventional T2T trial exploring the differences of reductions in magnetic resonance imaging (MRI) inflammation between patients who achieved ‘inactive disease’ *versus* no ‘inactive disease’ according to ASDAS. The TICOSPA trial showed no statistically significant improvement in the T2T group compared with the SOC group.^32^ Nevertheless, a positive trend for all outcomes in the T2T group as well as higher cost-effectiveness was observed.^32^ The treatment target in the TICOSPA trial was an ASDAS <2.1^32^ allowing also ‘low disease activity’ and not only ‘inactive disease’. The T2T recommendations for axial SpA, however, recommended ‘inactive disease’ as the preferred target and ‘low disease activity’ only as an alternative target.^8^ Consequently, the T2T concept in the TICOSPA trial might have reached a statistically significant benefit with a more stringent treatment target of ASDAS < 1.3. The currently ongoing AScalate trial has already adopted ASDAS-Q as disease activity score in the T2T arm for guiding therapeutic decisions due to the initial positive results for ASDAS-Q.^19^

A notable limitation of our study is the cross-sectional character. In view of the high sample size, the multicentric design and very similar results in comparison with our former study,^19^ however, we would not expect divergent results in a longitudinal analysis. Another possible limitation might be that all participating study centres were located in Berlin with 3.7 million inhabitants.^34^ Again, we would not expect substantially different results if some study centres were located in other cities, as both specialized academic centres and different private outpatient settings were included, and different local laboratories, including central laboratories from multi-national clinical trials were used.

Aranda-Valera *et al.*^35^ and Ortolan *et al.*^36^ proposed alternative formulas to calculate ASDAS out of BASDAI parameters/values and CRP values. These formulas are especially meaningful for retrospective research projects in study cohorts prior to the introduction of the ASDAS and reflect the need of the ASDAS in present axial SpA research to evaluate disease activity. The current study provides a method to calculate ASDAS values in prospective clinical studies or in clinical routine, when using routine laboratory CRP would lead to a significant delay for the calculation of the ASDAS.

According to these and former study results,^19^ ASDAS-Q combines the advantages of being quickly available while providing the accuracy of the conventional ASDAS-CRP in distinguishing between high and low disease activity with corresponding prognostic significance. These advantages cannot be achieved to the same extent by ASDAS-ESR as its time delay constitutes at least 1 h after blood collection and a relevant number of patients would be assigned to another disease activity category as with ASDAS-CRP.

In conclusion, ASDAS-Q has been validated in a large, multicentre axial SpA cohort including patients with the whole spectrum of axial SpA covering all disease stages and treatment modalities. Consequently, ASDAS-Q can help to facilitate the widespread use of ASDAS in clinical routine, to gain further direct evidence for the T2T concept in clinical trials, and to accelerate the implementation of the T2T concept in clinical routine. As a next step, the sensitivity to change of the ASDAS-Q will be evaluated in a multicentric and longitudinal trial, which is currently ongoing.

## Supplemental Material

sj-docx-1-tab-10.1177_1759720X221085951 – Supplemental material for Validation of the ASDAS with a quick quantitative CRP assay (ASDAS-Q) in patients with axial SpA: a prospective multicentre cross-sectional study Click here for additional data file. Supplemental material, sj-docx-1-tab-10.1177_1759720X221085951 for Validation of the ASDAS with a quick quantitative CRP assay (ASDAS-Q) in patients with axial SpA: a prospective multicentre cross-sectional study by Fabian Proft, Julia Schally, Henning Christian Brandt, Jan Brandt-Juergens, Gerd Rüdiger Burmester, Hildrun Haibel, Henriette Käding, Kirsten Karberg, Susanne Lüders, Burkhard Muche, Mikhail Protopopov, Judith Rademacher, Valeria Rios Rodriguez, Murat Torgutalp, Maryna Verba, Silke Zinke and Denis Poddubnyy in Therapeutic Advances in Musculoskeletal Disease
