# Supplementary material for: Validation of the ASDAS with a quick quantitative CRP assay (ASDAS-Q) in patients with axial SpA: a prospective multicentre cross-sectional study
Source: Ther Adv Musculoskelet Dis. 2022 Mar 30;14:1759720X221085951. doi: 10.1177/1759720X221085951 (PMC8972926; doi:10.1177/1759720X221085951)
Supplement: sj-docx-1-tab-10.1177_1759720X221085951 – Supplemental material for Validation of the ASDAS with a quick quantitative CRP assay (ASDAS-Q) in patients with axial SpA: a prospective multicentre cross-sectional study [file sj-docx-1-tab-10.1177_1759720X221085951.docx]

**Supplementary data**

Supplementary Table S1: Patients with different disease activity categories by ASDAS-Q and ASDAS-CRP.

| **ASDAS-CRP** | **ASDAS-Q** | **ASDAS-ESR** | **Routine laboratory CRP value (mg/l)** | **qCRP value (mg/l)** | **ESR value (mm/h)** |
| --- | --- | --- | --- | --- | --- |
| 1.1 (ID) | 1.3 (LDA) | 1.4 (LDA) | 2.1 | 3.4 | 10 |
| 1.1 (ID) | 1.4 (LDA) | 0.9 (ID) | 2.0 | 3.8 | 2 |
| 1.7 (LDA) | 2.2 (HDA) | 2.3 (HDA) | 2.0 | 5.7 | 20 |
| 1.8 (LDA) | 2.1 (HDA) | 2.1 (HDA) | 8.3 | 13.0 | 30 |
| 1.9 (LDA) | 2.1 (HDA) | 1.5 (LDA) | 11.5 | 15.0 | 14 |
| 2.0 (LDA) | 2.1 (HDA) | 2.0 (LDA) | 3.6 | 4.3 | 10 |
| 2.0 (LDA) | 2.1 (HDA) | 2.1 (HDA) | 3.8 | 4.6 | 14 |
| 2.0 (LDA) | 2.1 (HDA) | 2.0 (LDA) | 3.4 | 4.2 | 12 |
| 2.0 (LDA) | 2.1 (HDA) | 2.1 (HDA) | 11.6 | 14.0 | 28 |

ASDAS = Ankylosing Spondylitis Disease Activity Score, CRP = C-reactive protein, ESR = erythrocyte sedimentation rate, HDA = high disease activity, ID = inactive disease, LDA =low disease activity, qCRP = quick quantitative CRP.
